# Reporting issues with systematic reviews and meta-analyses

**DOI:** 10.1308/rcsann.2025.0034

**Published:** 2025-06-17

**Authors:** V Sahni

**Affiliations:** All India Institute of Medical Sciences (AIIMS), India

I write further to a recent paper in *The Annals* entitled ‘Revisional bariatric surgery following sleeve gastrectomy: a meta-analysis comparing Roux-en-Y gastric bypass and one anastomosis gastric bypass’.^1^

The methods section of the paper appears to provide the search strategy under the heading for ‘eligibility criteria’. This section should ideally contain the inclusion and exclusion criteria utilised for the study with a separate section detailing the search strategy that is identified as such. Further, the section on ‘search strategy’ appears to describe the screening process utilised for the articles retrieved from the search conducted.

Such issues will inevitably affect the rigor and quality of the report.
